# Identification of a Pain-Specific Gene Expression Profile for Pediatric Recurrent Abdominal Pain

**DOI:** 10.3389/fpain.2021.759634

**Published:** 2021-11-05

**Authors:** Adam B. Willits, Victoria Grossi, Nicole C. Glidden, Jeffrey S. Hyams, Erin E. Young

**Affiliations:** ^1^Neuroscience Program, KU Medical Center, Kansas City, KS, United States; ^2^Division of Digestive Diseases, Hepatology, and Nutrition, Connecticut Children's Medical Center, Hartford, CT, United States; ^3^Genetics and Genome Sciences, University of Connecticut School of Medicine, Farmington, CT, United States; ^4^Department of Anesthesiology, KU Medical Center, Kansas City, KS, United States

**Keywords:** irritable bowel syndrome, functional abdominal pain, gene expression, recurrent abdominal pain, genes

## Abstract

**Objectives:** Functional Abdominal Pain (FAP) and Irritable Bowel Syndrome (IBS) are common recurrent abdominal pain diagnoses with the hallmark, lack of inflammation. To identify a biological signature for IBS/FAP in the colon, this study used genetic profiling to uncover gene expression changes associated with IBS/FAP and abdominal pain.

**Methods:** Patients (8 to 17 years) newly diagnosed with IBS or FAP were enrolled in the study. At diagnostic colonoscopy, three rectal biopsies were collected, and gene expression analysis was performed using a Qiagen PCR Array. Relative fold difference in gene expression for 84 pain-associated genes was calculated using the 2-ΔΔ Cq method compared with pain-free controls. Factors affecting pain burden (Pain Burden Interview; PBI) were analyzed, including age, sex, rectal inflammation, and gene expression. Data were analyzed using multiple stepwise linear regression and 2-tailed t tests (*P* ≤ 0.05).

**Results:** Of the 22 total patients in the study, 19 were diagnosed with either IBS-Constipation (frequency of 5.26%), IBS-Diarrhea (47.37%), IBS-Mixed (10.53%), or FAP (36.84%). IBS/FAP patients reported significantly higher pain burden at the time of diagnosis compared to pain-free controls (*p* < 0.001), as well as significantly higher abdominal pain (*p* = 0.01). Of the 84 genes, expression of *GRIN1* (*p* = 0.02), *MAPK3* (*p* = 0.04), *P2X4* (*p* = 0.04), and *PTGES3* (*p* = 0.02) were all significantly associated with PBI score.

**Discussion:** Abdominal pain associated with IBS/FAP in pediatric patients may be linked to the expression of *GRIN1, MAPK3, P2X4*, and *PTGES3, pointing to potential novel* therapeutic targets for management of recurring abdominal pain.

## Introduction

Functional abdominal pain-not otherwise specified (FAP) and irritable bowel syndrome (IBS) are two common pediatric diagnoses characterized by recurrent abdominal pain (RAP) with no obvious organic cause (1). RAP is reported in 2–4% of all pediatric clinical visits and accounts for up to 25% of gastroenterology referrals (2, 3). Recurrent abdominal pain in school-aged children ranges from 11 to 38% with upwards of 3% reporting pain as a daily occurrence (4–6). RAP is a broad term encompassing various diagnoses where the underlying cause of abdominal pain is unclear but where pain is reported as frequent and very often impairs daily activities (e.g., school attendance, participation in extracurricular activities, etc.) (5). When compared to healthy peers, children with RAP are more likely to report other painful conditions [e.g., headaches; (7)] as well as increased depressive symptoms and social isolation (6), all of which contribute to reduced quality of life as seen in other groups with chronic health conditions (8). While diagnosis of FAP and IBS are based on specific clinical symptom criteria (9), the mechanisms underlying RAP, more generally, and those contributing to its persistence are not well understood. This knowledge gap poses problems with clear diagnosis, e.g., due to a lack of biochemical diagnostic biomarkers, and with the evidence needed to implement precision pain management strategies.

In both IBS and FAP, there can be hypersensitivity to normal function/stretching of the bowel resulting from reduced threshold for stretch stimuli, increased sensory afferent excitability (peripheral sensitization), and/or facilitation of central nervous system transmission of pain signals (central sensitization) (10). In other diseases and pathological conditions, peripheral sensitization often results from the inflammatory response to tissue damage, but since there is no occult inflammation or clearly identified organic disease in IBS/FAP, the cause of hypersensitivity remains unclear (10, 11). However, persistent, subclinical inflammation from a previous infection or other unresolved inflammatory process has been implicated in the etiology of IBS/FAP and could explain the symptoms of recurrent abdominal pain and visceral hypersensitivity (12, 13). However, traditional diagnostic techniques (i.e., histology) suggest no abnormalities in those with IBS/FAP while other techniques (i.e., immunohistochemical, ultrastructural analyses) suggest subtle changes in the bowel that may or may not be related to pain, *per se* (14).

In addition to paucity in the literature surrounding the etiology of abdominal pain in IBS and FAP, there are few clinical interventions available for the treatment of persistent abdominal pain that do not also disrupt bowel habits. Opioids are often given for acute episodes of abdominal pain and/or when pain escalates suddenly, but these medications cause constipation which can intensify IBS pain even in those patients with diarrhea predominant IBS (15) and therefore are contraindicated in patients with IBS/FAP (15, 16). Other current IBS treatments included laxatives and antispasmodics in the case of constipation (IBS-C) and anti-diarrheal medications for diarrhea (IBS-D), with even fewer treatment options for mixed/alternating (IBS-M) (17, 18). These treatments can temporarily reduce other IBS symptoms in the short term, but they have been shown to have no significant effect on visceral hypersensitivity or abdominal pain (15).

FAP is routinely used as a synonym for IBS since both are functional disorders of the gut with slight similarities in clinical presentation. However, FAP is a separate gastrointestinal syndrome characterized by recurring abdominal pain with no significant alterations to gastrointestinal motility or function (19, 20) which makes it even more resistant to treatment. For both IBS and FAP, the primary and most debilitating symptom is pain, and with no organic disease to treat in order to reduce that pain, patients report significant reduction in quality of life (21), highlighting the need for precision pain management strategies to address abdominal pain in these two populations.

While IBS/FAP is characterized by a lack of occult disease or pathology in the bowel, the identification of a biological signature or phenotype could be helpful in identifying novel therapeutic targets for abdominal pain in these conditions. By conducting a tissue-specific gene profiling study, it is possible to determine changes in colon tissue gene expression that are correlated with gastrointestinal disorders (22). This may determine how genetic components of IBS/FAP result in changes in enteric nervous system electrical excitability and could contribute to new mechanisms to therapeutically reduce abdominal hypersensitivity (23). In order to bridge the gap between IBS/FAP-associated abdominal pain and genetics, our group compared gene expression from pediatric patient colon biopsies and determined genetic correlations with pain burden surveys.

## Methods

### Patient Population

Subjects between the ages of 8 and 17 years who were undergoing diagnostic colonoscopy for differential diagnosis of Inflammatory Bowel Disease were recruited for the ALLAY Study (Assessing Risk Factors for Abdominal Pain in Children with Inflammatory Bowel Disease). Patients undergoing colonoscopy for reasons other than abdominal pain (painless rectal bleeding and persistent, painless nausea) were recruited for a pain-free control population. A diagnosis of IBS and FAP (not otherwise specified) by well-established Rome IV criteria was required for further inclusion in the present analysis (9). The sample size was based on the total number successfully recruited from an urban children's medical center in a 12-month period. We enrolled only newly diagnosed patients as they were naïve to treatment, naïve to diagnosis, and did not have a history of pain-related diagnoses and were seeking treatment for the onset of abdominal pain with or without other symptoms. Examination of the EMR and patient self-report were used to exclude patients who had previously been diagnosed with another chronic pain disorder (fibromyalgia, migraine, etc.). It is possible that these disorders would emerge overtime as chronic pain conditions often emerge during adolescence and often co-occur. However, at the time of recruitment and inclusion in our study, participants were excluded if they had a prior diagnosis of a chronic pain condition. Other exclusion criteria included prior abdominal surgery unrelated to diagnosis, active gastrointestinal infection at the time of diagnosis (e.g., *Helicobacter pylori, Clostridium difficile*), other comorbidities that may affect abdominal pain (e.g., familiar Mediterranean fever), or diagnosis of Crohn's disease or Ulcerative Colitis based on pathology. Patients and parents were required to read and speak English. Written informed consent was provided by all parents/guardians, and written assent was obtained from the subjects. Participants were reimbursed $50 for completion of all survey measures and biological sample collection.

Approval for this study was granted through the Institutional Review Board at Connecticut Children's Medical Center.

### Patient Outcomes

#### Experimental Design

*Irritable Bowel Syndrome* diagnosis was based on established Rome IV criteria (9), i.e., recurrent abdominal pain occurring a minimum of 1 day per week for the prior 3 months and associated with one or more of (1) increased pain related to defecation, (2) change in stool frequency and (3) change in form (appearance) of stools. IBS subtype diagnosis was based on patient report of predominant alteration in bowel habit [i.e., constipation (IBS-C), diarrhea (IBS-D), or mixed/alternating (IBS-M)]. IBS diagnosis was based on ROME IV criteria (9). *Functional Abdominal Pain-not otherwise specified* (FAP) diagnosis (Rome IV) was defined by the presence of episodic or continuous abdominal pain, not dependent on other physiological events (eating, menses, etc.), and occurring at least four times per month for a minimum of 2 months prior to diagnosis. The Rome IV criteria definitively categorize functional disorders, including FAP and IBS, as occurring along a continuum rather than as distinct disorders. Given that our goal of identifying potential relationships between patient characteristics, gene expression profiling within the colorectum and abdominal pain explicitly, we included both diagnoses characterized by significant pain occurring over months prior to diagnosis. For those diagnosed with IBS and FAP, clinical information such as age, sex, disease type (IBS-D, IBS-C, IBS-M, or FAP) (9), pain burden at the time of diagnosis (assessed by Pain Burden Interview), patient self-report pain-related psychological measures, and degree of rectal inflammation at the time of colonoscopy were collected via chart review.

#### Pain Burden

All patients enrolled completed the Pain Burden Interview (PBI) within 1 month of their initial/diagnostic colonoscopy and were asked to complete it in regards to their abdominal pain. The PBI is a 7-item functional assessment measure with forced choice answers (none, a few, some, many, and every). It yields a numeric score, with a higher score indicating increased pain burden. The score ranges from 0 (no burden) to 28 (severe burden) and has been validated for use within the pediatric and/or adolescent populations with various pain-related conditions (24).

#### Other Pain-Related Outcomes

All patients enrolled also completed the Pain Frequency-Severity-Duration Scale (PFSDS), Adolescent Pediatric Pain Tool (APPT), Pain Catastrophizing Scale—Child Version (PCS-C), and Children's Somatization Inventory-24 (CSI-24) within 1 month of their initial/diagnostic colonoscopy; these questionnaires are validated for the general pediatric and/or adolescent population (25–28). The PFSDS is designed to assess pain frequency, severity, duration, and how pain intensity relates to pain intrusiveness (25). The APPT is a self-report measure to breakdown the intensity and location of pain (29). The PCS-C is a trait measure that assesses the extent to which children worry when they are in pain. A total score is calculated, in addition to three other subscores assessing rumination, magnification, and helplessness (26). The CSI-24 is a 24-item, self-report questionnaire to assess children's perception of somatic symptoms (28).

### Biospecimen Collection and Gene Expression Analysis

At the time of diagnostic colonoscopy, 3 rectal biopsies were collected for study purposes, placed in 200 μL RNA*later* (Thermo Fisher Scientific, Waltham, Massachusetts), and refrigerated immediately. Pain-free patient biopsies were collected adjacent to the site of rectal bleeding to insure healthy tissue for the control group. These samples were then frozen at −80°C for subsequent batch processing. Total RNA was extracted from each sample (three biopsies/individual patient) using RNeasy Mini Kits (Qiagen, Hilden, Germany) according to manufacturer's instructions. The concentration of total RNA was determined using a biospectrophotometer (Eppendorf, Hamburg, Germany), and cDNA was produced from 500 ng of total RNA using iScript cDNA synthesis kits according to manufacturer's instructions (BioRad). Gene expression analysis was performed using Qiagen RT2 Profiler Neuropathic and Inflammatory Pain PCR Array. Each real-time quantitative PCR (qPCR) plate contains primers specific to 84 genes previously implicated in the pain response, housekeeping genes, and appropriate controls. Differential gene expression was calculated as fold-difference in expression using the 2^−ΔΔCq^ method relative to expression in the pain-free controls. Fold-difference in expression calculations are based on normalization using the average of the three most stable housekeeping genes (*GAPDH, ACTB*, and *B2M*). For each of the 84 “genes of interest” (GOI), the ΔC_q_ value was calculated as ΔCq = Cq,_GOI_−Cq,_HKG_. Thereafter, for each subject, the relative fold change in expression was calculated as 2^−ΔΔCq^, where Δ(ΔC_q_) = ΔC_q_,RAP–ΔC_q_,_Painless Control average_.

### Statistical Analysis

Statistical analysis was performed using multiple linear regression and 2-tailed *t-*test, utilizing IBM Statistical Package for the Social Science (SPSS, v24, IBM). Only participants who completed all survey measures and tissue biopsy collection/quality control for samples successfully were included. Missing data were not extrapolated from group data. Stepwise linear regressions were conducted to examine associations between expression of genes implicated in the conduction of pain and pain outcomes. Demographic variables shown previously to be associated with increased pain burden in IBS/FAP (age, sex) (30–33) were loaded into all regressions as factor 1. Normalize expression for each gene was entered as factor 2 in linear regression analyses to independently evaluate the strength of each gene as a biomarker for abdominal pain factor. Genes that were found to have a statistically significant association to PBI in the regression analyses were further evaluated using a 2-tailed *t* test to compare expression of the candidate genes for patients with no pain (PBI ≤ 2) to patients with pain (PBI > 2). All patients in the RAP group reported PBI scores >2. A *p* ≤ 0.05 was considered statistically significant for all analyses. It was decided prior to conducting analyses that we would not adjust the *p* value for multiple corrections, in part because we had no a priori hypotheses regarding relationships between the independent variables and assumed that multiple genes could explain small but significant portions of the variance; in addition, we had an explicit goal of leveraging this unique data set to generate hypotheses for subsequent, hypothesis-driven mechanistic testing in preclinical and/or clinical populations. While it could be argued that a conservative correction of the *p* value for multiple comparisons is the most appropriate method for this study, this comes with the increased risk for Type II errors of “false negatives.” Given the high potential for translation into precision interventions for children with recurrent abdominal pain, the relative risk of missing a clinically relevant relationship was deemed of greater importance than the risk of Type I errors that would be resolved with future studies.

## Results

### Sample Characteristics

**Figure 1:**
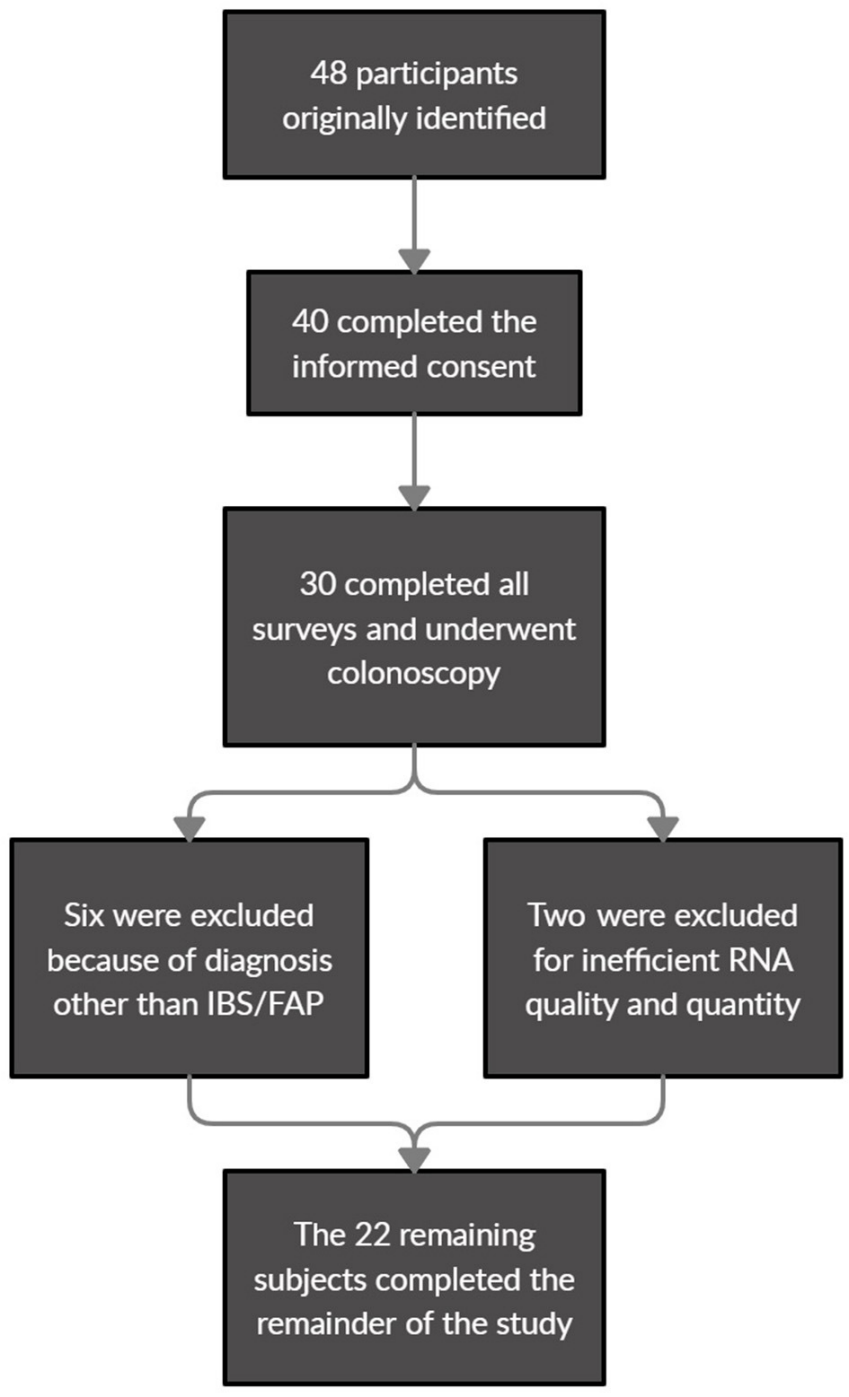
Patient eligibility identification, recruitment, and exclusion flowchart for final analysis.

A total of 22 pediatric patients were included in the final analysis in this study. Forty eight participants were initially identified as eligible for the study with 40 completing the informed consent for recruitment. Of the 30 participants who ultimately completed all surveys and underwent diagnostic colonoscopy, six were ultimately excluded by diagnosis with a disorder other than IBS/FAP (*Helicobacter pylori*, Inflammatory bowel disease) and an additional two participants were excluded when their biological samples failed to meet the RNA quality and quantity benchmarks (Figure 1). The surveys covered topics ranging from pain burden to other various psychological measures, while the tissue collection from a colonoscopy was used for gene expression analysis. The mean (±SEM) age of the patient cohort was 14.09 (±0.48), and 10 of the 22 (45.5%) were male. The patients that reported pain were diagnosed with either IBS-C (frequency of 5.26%), IBS-D (47.37%), IBS-M (10.53%), or FAP (36.84%). No patients were found to have identifiable rectal inflammation at the time of colonoscopy. Full descriptive statistics and clinical surveys results are displayed in Table 1.

**Table 1:** Patient cohort descriptive statistics and clinical survey measures.

	**Control**	**IBS/FAP**
Total patients	3	19
Age	13 (±2.08)	14.26 (±0.73)
M:F	33%	47%
**Diagnosis**		
IBS-C	-	5.26%
IBS-D	-	47.37%
IBS-M	-	10.53%
FAP	-	36.84%
**Pain-related measures**		
CSI-24	8.67 (±7.69)	27.37 (±2.84)
PCS-C	13 (±9.29)	26.68 (±2.64)
Rumin	7 (±3.79)	11.05 (±0.89)
Magnf	1.33 (±1.33)	4.79 (±0.72)
Helpless	4.67 (±4.67)	10.84 (±1.29)
**Pain measures**		
PBI	0 (±0)	12.37 (±1.10)
APPT		
Tot BSA	0 (±0)	516.16 (±107.50)
Tot # areas	0 (±0)	2.68 (±0.36)
Abd BSA	0 (±0)	301.11 (±41.76)
Abd # areas	0 (±0)	1.79 (±0.12)
**PFSD**		
# of days	0 (±0)	10.56 (±0.86)
Lvl pain	0 (±0)	5.86 (±0.41)
Hrs pain	0 (±0)	2.63 (±0.34)
Worst pain	0 (±0)	7.71 (±0.33)
Hrs worst pain	0 (±0)	1.79 (±0.25)
Comp-worst	0 (±0)	14.5 (±2.56)

### Patient Reported Pain

**Figure 2:**
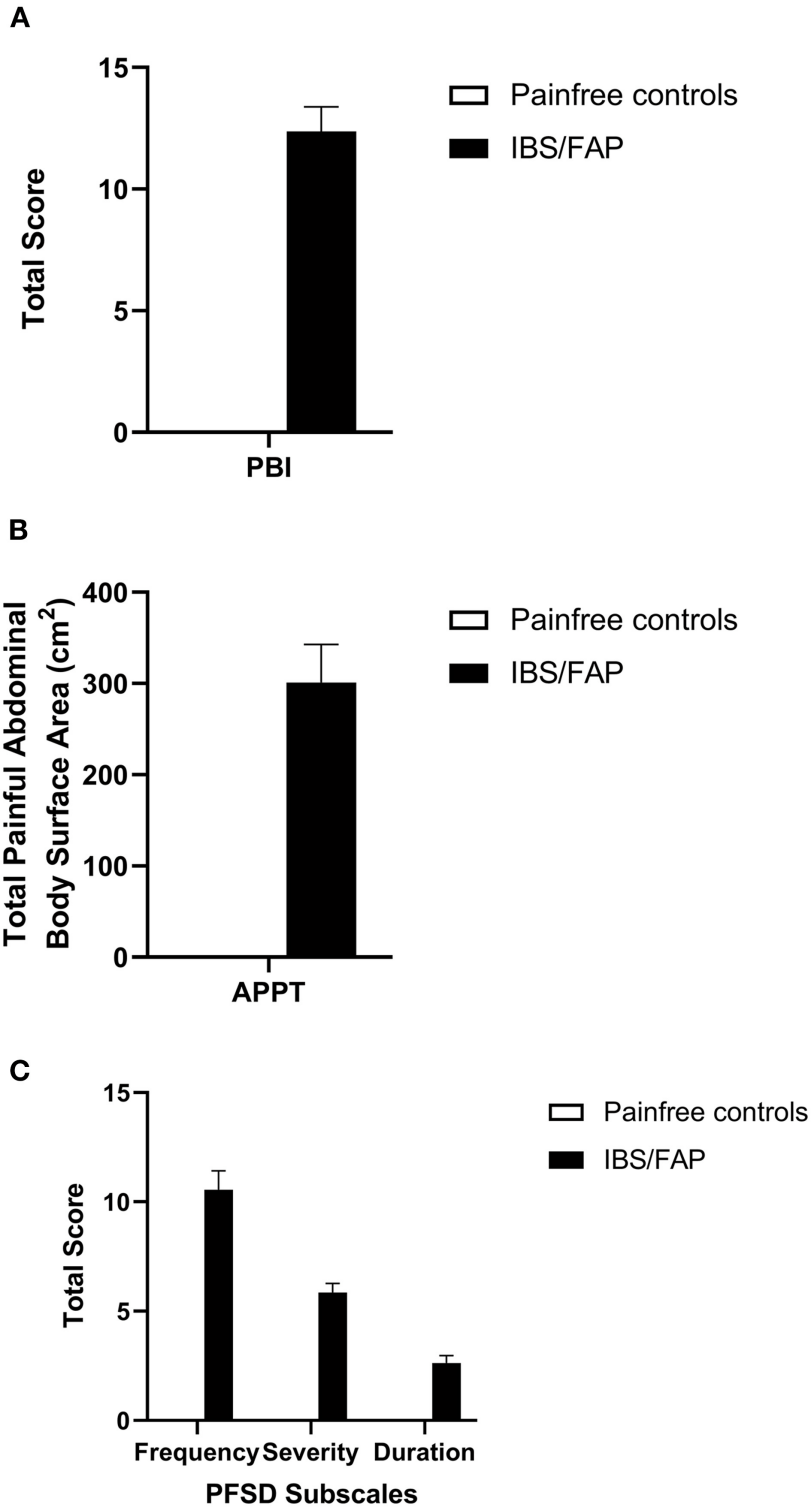
**(A)** IBS/FAP patients reported significantly higher pain burden at the time of diagnosis compared to pain-free controls (*p* < 0.000). **(B)** IBS/FAP patients reported significantly higher abdominal BSA (*p* = 0.011), which was also significantly correlated with PBI scores using a Pearson correlation coefficient (*p* = 0.020). **(C)** The PFSD subscales, Frequency (*p* = 0.000), Severity (*p* = 0.000), and Duration (*p* = 0.007), were all significantly different between IBS/FAP patients and pain-free control patients.

All patients diagnosed with IBS/FAP reported abdominal pain at the time of diagnosis. Three patients reporting undergoing colonoscopy for painless rectal bleeding also completed surveys and tissue collection as pain-free controls. The mean (±SEM) PBI score for IBS patients was 12.37 (±1.01) (Figure 2A) while controls reported 0 (±0). An independent sample *t* test confirmed that IBS/FAP patients reported significantly higher pain burden at the time of diagnosis compared to pain-free controls (*p* < 0.001).

### Pain-Related Measures

**Figure 3:**
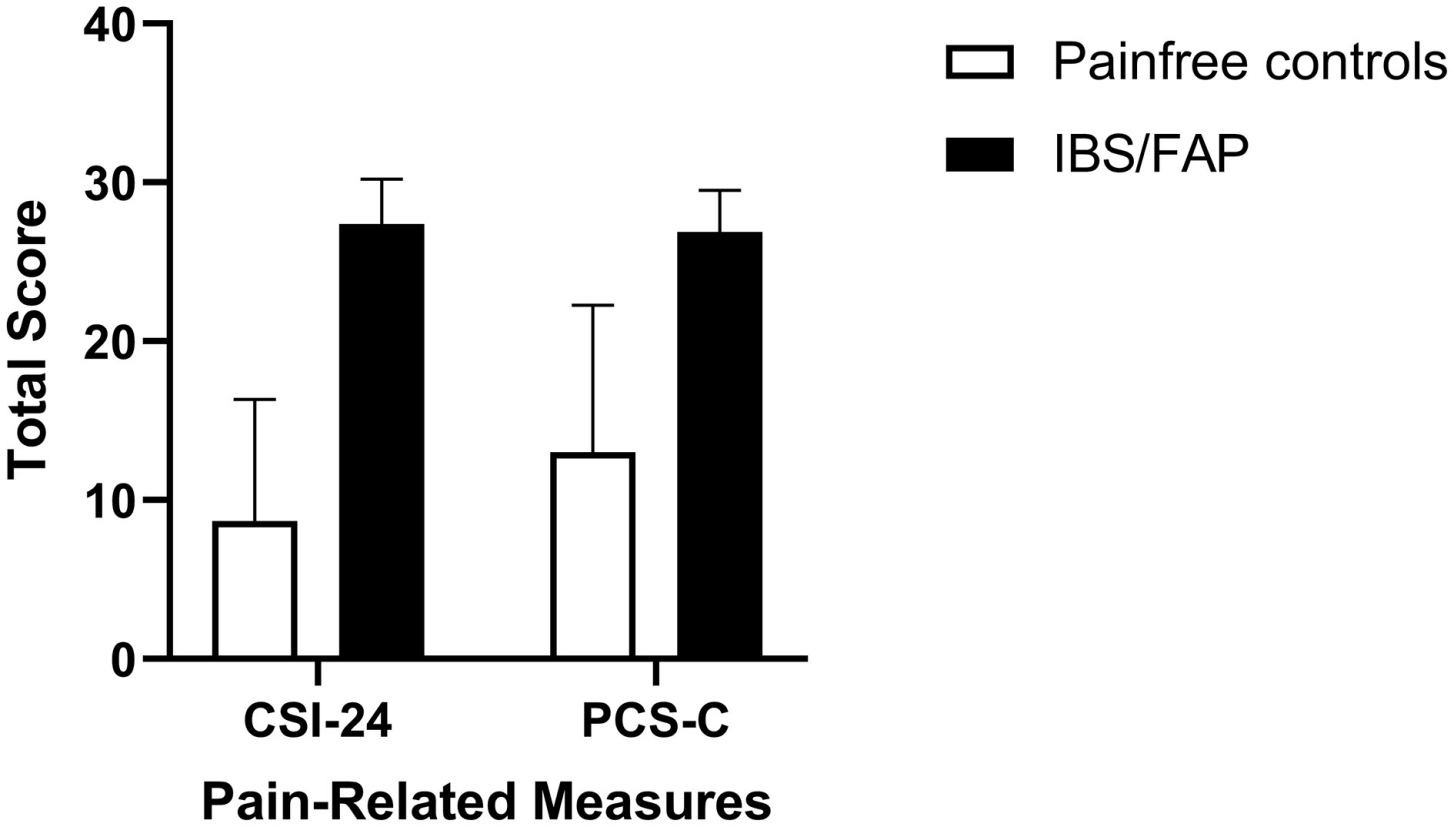
The average level of somatization (CSI-24) and pediatric catastrophizing (PCS-C) in newly diagnosed IBS/FAP patients compared to pain-free controls. Somatization (*p* = 0.026), but not catastrophizing (*p* = 0.083), was significantly higher in IBS/FAP patients compared to pain-free controls.

The average level of somatization (CSI-24) in newly diagnosed IBS/FAP patients was 27.37 (±2.84) and for pain-free controls was 8.67 (±7.69) (Figure 3). Independent sample *t* test results were significantly different between pain-free subjects and pain groups (*t* = −2.411, *p* = 0.03). The PCS-C total score for the IBS/FAP group was 26.68 (±2.64) while the pain-free control group reported lower PCS-C total scores of 13 (±9.29) (Figure 3). An independent sample *t* test showed nonsignificant results for PCS-C (*t* = −1.826, *p* = 0.08). The average rumination, magnification, and helplessness subscales (PCS-C) for IBS/FAP patients were 11.05 (±0.89), 4.79 (±0.72), and 10.84 (±1.29), respectively, compared to pain-free control subjects' average scores of 7.0 (±3.79), 1.33 (±1.33), and 4.67 (±4.67). Independent sample *t* tests showed that neither rumination (*t* = −1.544, *p* = 0.14), magnification (*t* = −1.815, *p* = 0.08), nor helplessness (*t* = −1.684, *p* = 0.11) were significantly different between IBS/FAP and pain-free controls.

Based on APPT scores, the abdominal BSA and number of painful abdominal areas for pain-free control subjects were both 0 (±0), while the IBS/FAP group reported elevated scores of 301.11 (±41.76) and 1.79 (±0.12) for those two measures, respectively (Figure 2B). An independent sample *t* test confirmed that IBS/FAP patients reported significantly higher abdominal BSA (*t* = −2.807, *p* = 0.01) and number of painful abdominal areas (*t* = −4.620, *p* < 0.001) compared to pain-free controls. PFSD scores evaluating the number of days (frequency), level of pain (severity), hours of pain (duration), worst pain, and hours of worst pain indicated elevated scores for the IBS/FAP patients of 10.56 (±0.86), 5.86 (±0.41), 2.63 (±0.34), 7.71 (±0.33), and 1.79 (±0.25), respectively, compared to scores of 0 (±0) on all measures for the pain-free controls (Figure 2C). The independent sample *t* test results were, respectively, (*t* = −4.903, *p* < 0.001), (*t* = −5.569, *p* < 0.001), (*t* = −2.980, *p* = 0.007), (*t* = −9.045, *p* < 0.001), (*t* = −2.800, *p* = 0.01), and (*t* = −5.654, *p* < 0.001).

### Correlations Between PBI and Pain-Related Measures

Pearson correlations between individual patient pain burden (PBI) and pain-related outcomes were performed. CSI-24 was significantly correlated with pain burden (r_p_ = 0.448, *p* = 0.04) as well as PCS-C (r_p_ = 0.501, *p* = 0.02). Rumination was not significantly correlated with PBI (r_p_ = 0.397, *p* = 0.07), but magnification (r_p_ = 0.462, *p* = 0.03) and helpless (r_p_ = 0.512, *p* = 0.02) were significantly correlated with pain.

PBI scores were significantly correlated with the number of abdominal pain areas reported using the APPT (r_p_ = 0.502, *p* = 0.02), but not with the total abdominal BSA (r_p_ = 0.295, *p* = 0.18). Finally, all PFSD subscale measures were significantly correlated with PBI: the number of days with pain (r_p_ = 0.827, *p* < 0.001), the level of pain (r_p_ = 0.861, *p* < 0.001), the hours of pain (r_p_ = 0.659, *p* < 0.001), worst pain (r_p_ = 0.880, *p* < 0.001), and the hours of worst pain (r_p_ = 0.661, *p* < 0.001).

To continue to understand the relationship between these survey measures and pain, we conducted independent sample *t* tests for each of these pain-related measures comparing the pain-free control group to the IBS/FAP group. The only pain-related measure that was significantly different between the IBS/FAP and pain-free groups was CSI-24 (*t* = −2.411, *p* = 0.03). All other scores had non-significant *p* > 0.05. Since CSI-24 was significant, it was included later in the linear regressions for gene expression analysis.

### Associations Between Demographics, Pain-Related Measures and Pain

We evaluated potential associations between demographic factors and pain burden using stepwise linear regression in patients newly diagnosed with IBS or FAP. Age and sex were not associated with pain burden in IBS/FAP patients, however we included both factors in subsequent analyses as prior research suggests that age and/or sex play a role in pain outcomes in pediatric populations. In addition, CSI-24 was included as a second step in the regression since it was identified through individual sample *t* tests (*t* = −2.411, *p* = 0.03) to be a potential contributing factor for pain but was unlikely to be associated with colon-specific gene expression.

### Identification of Pain-Relevant Candidate Genes

**Figure 4:**
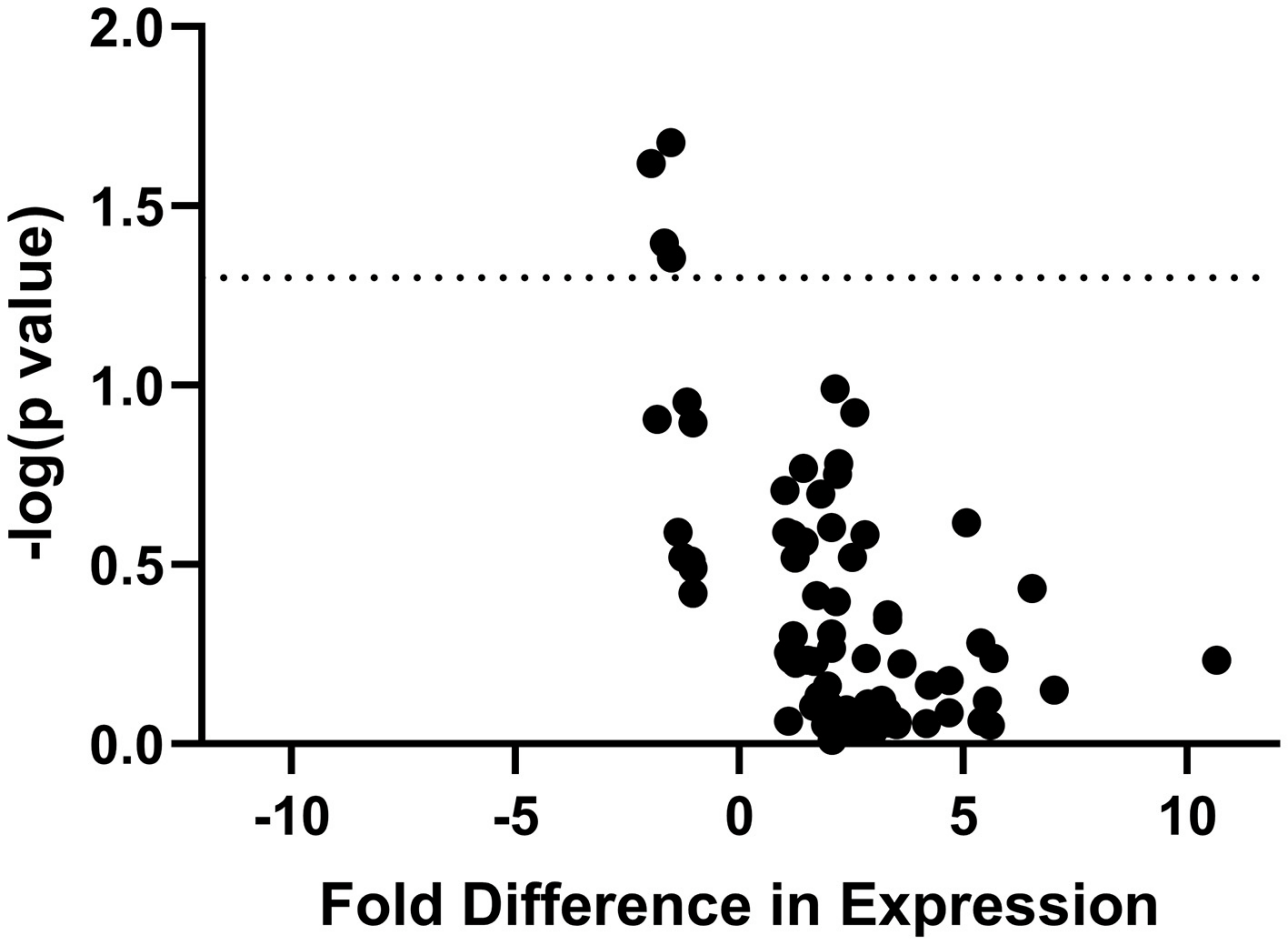
“Volcano Plot” of statistical significance (-log[p value] of association with PBI score) against fold difference in expression for all IBS/FAP patients. The figure demonstrates the four of 84 genes (*GRIN1, MAPK3, P2X4, PTGES3*) whose expression is significantly associated with pain burden in the patient population.

Of the 84 pain-relevant genes that were analyzed, only four showed significant association with pain burden in stepwise linear regression: *GRIN1* (Δr^2^ = 0.205, ΔF = 6.429, *p* = 0.02), *MAPK3* (Δr^2^ = 0.168, ΔF = 4.941, *p* = 0.04), *P2X4* (Δr^2^ = 0.162, ΔF = 4.717, *p* = 0.04), and *PTGES3* (Δr^2^ = 0.198, ΔF = 6.146, *p* = 0.02) (Figure 4). Since we discovered through the stepwise linear regressions that *GRIN1, MAPK3, P2X4*, and *PTGES3* are significantly associated with pain burden, we compared expression of the genes between pain and pain-free subjects, individually. We calculated individual sample *t* tests for *GRIN1* (*t* = 1.449, *p* = 0.16), *MAPK3* (*t* = 1.369, *p* = 0.19), *P2X4* (*t* = 1.329, *p* = 0.20), and *PTGES3* (*t* = 2.017, *p* = 0.06). While they were each significantly associated with PBI score, none of the prioritized candidate genes was differentially expressed between patients reporting significant pain and pain-free controls, though *PTGES3* approached significance.

## Discussion

The biological mechanisms underlying RAP are not well understood, and this paucity in the literature translates to a lack of evidence-based interventions targeting pathological pain processes within the bowel. Visceral hypersensitivity is a characteristic clinical marker for IBS/FAP, yet the mechanisms remain incompletely understood. Uncovering relationships between individual differences in bowel-specific gene expression and abdominal pain could be a steppingstone to unraveling the mystery of IBS/FAP pathogenesis (34, 35) and provide insight into novel pain-specific treatment targets. For the present study, we analyzed the expression of 84 genes implicated in the transduction, maintenance, and modulation of pain, and the expression of four genes, *GRIN1, MAPK3, P2X4*, and *PTGES3*, was associated with pain burden in pediatric RAP patients. This mirrored the results of a recent systems biology-based study where this same set of genes, among others, was found to be enriched in patients with both acute and persistent post-surgical pain (36), adding further support to their potential role in pain susceptibility.

Our prior work focused on patients with active inflammation who met diagnostic criteria for Inflammatory Bowel Diseases (Crohn's Disease, Ulcerative Colitis, IBD-Unclassified). In the current manuscript, we focused entirely on the patients whose pain was unrelated to another diagnosis and occurred exclusively in the absence of inflammation (as required for RAP/IBS/FAP diagnoses). Given that our prior work and the work of others supports separate genetic mechanisms of risk for inflammatory pain and pain occurring in the absence of inflammation, whether spontaneous or elicited, we separated these participants into two separate analyses. While these data were collected in parallel, the difference in the source and nature of the pain points to different sources of abdominal pain. To this end, prior work from our group and others indicates separate genetic “sources” for pain due to inflammation compared to other sources. Our previous subanalysis of IBD participants highlighted potential roles for TRPV3, PTGS2, and MAPK14 in the IBD-related abdominal pain. The present data support the separation of these two classes of patients as there were no common genes of interest for new onset active Inflammatory Bowel Disease is distinct from that in IBS/FAP based on our analyses. As with most chronic health conditions, IBS/FAP is likely a complex interaction of multigenic risk and environmental factors and the present study points to four high priority candidate genes (*GRIN1, MAPK3, P2X4*, and *PTGES3*) where expression corresponds to pain severity.

*Glutamate receptor subunit zeta-1 (GRIN1)* encodes the GluN1 protein, one of three subunits of the N-methyl-D-aspartate (NMDA) receptor, a transmembrane-spanning, ion channel that binds glycine and glutamate ligands and is permeable to sodium, potassium, and calcium (37). This NMDA receptor-dependent cation movement across the cellular membrane is vital to many cellular functions including transmission of action potentials (38). Human neurological diseases (i.e., epilepsy, Parkinson's, intellectual disabilities) have been linked to NDMA ion channel defects resulting from altered function, activity, or expression levels (38). Functional studies on NMDA receptor subunits suggest that an alteration in even one subunit can significantly alter receptor function. For example, it has been shown that when GluN2A is reduced in NMDA receptors, there is a concomitant decrease in sensitivity to negative modulators and prolonged deactivation, suggesting that decreased subunit functionality may lead to increase receptor activity (39). The present finding of a negative correlation between *GRIN1* expression in colorectal biopsies and pain burden is in line with this prior work. When GluN1 is active, the channel deactivates more rapidly (40), presumably resulting in increased cell excitability; a reduction in *GRIN1* expression could slow deactivation resulting in increased cellular activity, both spontaneous and evoked. *GRIN1* mRNA is highly expressed in nervous system cells with protein expression localized to the synapses. While the mRNA is also found in low levels in other tissue types, it does not appear to be expressed in digestive system cells (Human Protein Atlas). While the present study design does not allow for mechanistic evaluation of hyperexcitability within neuronal cells innervating the colon, this could be a possible explanation of why pediatric patients with lower *GRIN1* expression reported greater pain burden.

*PTGES3*, encoding the protein prostaglandin E synthase-3, is an enzyme that is constitutively expressed in the cytoplasm and functions to synthesize prostaglandin E_2_ (PGE_2_) (41). PGE_2_ is proinflammatory, and variations in function or expression are associated with various neurodegenerative diseases (i.e., Parkinson's, Alzheimer's, amyotrophic lateral sclerosis) (42). PGE_2_ is typically expressed in low levels that can be increased in response to inflammation (43). In the context of pain, PGE_2_ can directly activate E prostanoid (EP) receptors, a family of G protein coupled receptors, to stimulate an increase in intracellular calcium levels through G protein signaling (42–45). This calcium influx in sensory neurons promotes neurotransmitter release into synaptic junctions, ultimately increasing neuronal communication and perception of pain. Therefore, since *PTGES3* expression is associated with pain in the current cohort of RAP patients, this could be a mechanism through which it regulates the pain susceptibility seen in this population.

*MAPK3*, encoding the protein mitogen-activate protein kinase, is an extracellular-regulated protein kinase (ERK) playing a critical role in various cellular processes, cell cycle, stress responses, and apoptosis, through regulation of intracellular signal transduction (46). In addition, ERKs can translocate to the nucleus to phosphorylate and activate transcription factors to alter gene expression (47). *MAPK3* is downregulated in the dorsal root ganglion of sensory neurons in neuropathic pain (48), but has also been shown to be upregulated in colon tissues with Inflammatory Bowel Disease (IBD) (49). IBD is an inflammatory disease which will, more often than not, include patient reported pain as a symptom. It is interesting to note that our study shows a negative correlation between IBS and *MAPK3* expression, while prior work has shown that IBD severity is positively correlated with *MAPK3* expression. As with a number of other profiles of gene expression, it is possible that it is the dysregulation of MAPK3 that is problematic, so increases or decreases in expression could disrupt function.

*P2X4* is a member of the P2X family of ligand-gated ion channels, and encodes a purinergic receptor sensitive to extracellular ATP levels (50, 51). P2X4 receptors are expressed in diverse cell types and locations, including neurons of the central and peripheral nervous systems, as well as visceral and vascular smooth muscle (52). *P2X4* knockout mice do not develop pain hypersensitivity, while mice that express activated *P2X4* develop hypersensitivity and harbor an increase in PGE2 production (53). As discussed above, PGE2 is a proinflammatory mediator of hypersensitivity. Therefore, *P2X4* and *PTGES3* may work synergistically to increase PGE2 and, subsequently to contribute to hypersensitivity due to prior inflammation or subclinical inflammation in IBS/FAP patients.

A limitation to the study was that our control group was ultimately three patients since it is difficult to recruit pediatric patients who are receiving colonoscopies without abdominal pain. The reduced availability of pain-free control patients remains one of the challenges of studying the correlation between pain and gene expression in pediatric IBS/FAP patients. While we recognize that this study is underpowered and may be subject to the identification of false positives due to Type 1 errors, we contend that identifying tissue specific gene expression profiles associated with pediatric recurrent abdominal pain is novel and offers the opportunity for clinically relevant, exploratory hypothesis generation that could be incorporated into followup mechanistic analyses. An additional limitation to the study regards the fact that colon biopsies taken from patients are heterogeneous samples with multiple cell types (i.e., nerve terminal endings, muscle layers, mucosal cells). This poses difficulty in determining which specific cell type(s) harbor the pain-associated gene expression changes.

Much of the research in human subjects depends on analysis of circulating blood due to its ubiquity in the healthcare setting and its relative ease of collection. In this study, we were able to capitalize on the diagnostic colonoscopies/biopsies occurring as part of the standard of care to identify differences in gene expression within the colorectum that may play a role in generation or maintenance of persistent pain. Understanding the gene expression changes that support and maintain persistent bowel pain offers insights into pain specific treatment strategies where the current approach is largely to treat alterations in bowel habits/gastric motility to reduce pain indirectly. We have shown that patient tissue biopsies can be used in conjunction with pain report surveys to uncover correlations between disease and gene expression. We identified four genes that were correlated with IBS/FAP pain: *GRIN1, MAPK3, P2X4*, and *PTGES3*. These could be possible candidate targets for therapeutics, but more needs to be researched about the function of these genes in the context of IBS/FAP pain. In addition, this study shows the need for independent research into bowel pain, even though it may be associated with IBS/FAP symptoms. Pain may be a separate pathological process that can be targeted to improve quality of life in parallel with targeting of other symptoms, and future studies need to continue uncovering the pathology of pain and contribute to clinical management of pain.

## Data Availability Statement

The raw data supporting the conclusions of this article will be made available by the authors, without undue reservation.

## Ethics Statement

The studies involving human participants were reviewed and approved by Institutional Review Board, Connecticut Children's Medical Center. Written informed consent to participate in this study was provided by the participants' legal guardian/next of kin.

## Author Contributions

AW prepared the data, completed all analyses, drafted the manuscript with JH and EY, and incorporated revisions provided from coauthors. VG recruited participants, oversaw patient data entry and management, and collected biological samples. NG completed all sample preparation, cataloging, and storage as well as gene expression analysis. EY and JH developed the project, designed the study, and supervised all data collection. All authors contributed to the article and approved the submitted version.

## Funding

This work was funded by Startup funds (EEY) from University of Connecticut School of Nursing.

## Conflict of Interest

JH is on the advisory board for Janssen and Abbvie and a consultate to Pfizer, Roche, Celgene, Allergan, Lily, and Boehringer-Ingelheim. The remaining authors declare that the research was conducted in the absence of any commercial or financial relationships that could be construed as a potential conflict of interest.

## Publisher's Note

All claims expressed in this article are solely those of the authors and do not necessarily represent those of their affiliated organizations, or those of the publisher, the editors and the reviewers. Any product that may be evaluated in this article, or claim that may be made by its manufacturer, is not guaranteed or endorsed by the publisher.

## References

[1] Chiou E Nurko S. Management of functional abdominal pain and irritable bowel syndrome in children and adolescents. Expert Rev Gastroenterol Hepatol. (2010) 4:293–304. 10.1586/egh.10.28 20528117 PMC2904303

[2] Starfield B Katz H Gabriel A Livingston G Benson P Hankin J. Morbidity in childhood–a longitudinal view. N Engl J Med. (1984) 310:824–9. 10.1056/NEJM198403293101305 6700671

[3] Crushell E Rowland M Doherty M Gormally S Harty S Bourke B. Importance of parental conceptual model of illness in severe recurrent abdominal pain. Pediatrics. (2003) 112:1368–72. 10.1542/peds.112.6.1368 14654611

[4] Hyams JS Burke G Davis PM Rzepski B Andrulonis PA. Abdominal pain and irritable bowel syndrome in adolescents: a community-based study. J Pediatr. (1996) 129:220–6. 10.1016/S0022-3476(96)70246-9 8765619

[5] Saps M Seshadri R Sztainberg M Schaffer G Marshall BM Di Lorenzo C. Prospective school-based study of abdominal pain and other common somatic complaints in children. J Pediatr. (2009) 154:322–6. 10.1016/j.jpeds.2008.09.047 19038403

[6] Youssef NN Atienza K Langseder AL Strauss RS. Chronic abdominal pain and depressive symptoms: analysis of the national longitudinal study of adolescent health. Clin Gastroenterol Hepatol. (2008) 6:329–32. 10.1016/j.cgh.2007.12.019 18258491

[7] Stordal K Nygaard EA Bentsen BS. Recurrent abdominal pain: a five-year follow-up study. Acta Paediatr. (2005) 94:234–6. 10.1080/08035250410025104 15981760

[8] Youssef NN Murphy TG Langseder AL Rosh JR. Quality of life for children with functional abdominal pain: a comparison study of patients' and parents' perceptions. Pediatrics. (2006) 117:54–9. 10.1542/peds.2005-0114 16396860

[9] Schmulson MJ Drossman DA. What is new in Rome IV. J Neurogastroenterol Motil. (2017) 23:151–63. 10.5056/jnm16214 28274109 PMC5383110

[10] Feng B La JH Schwartz ES Gebhart GF. Irritable bowel syndrome: methods, mechanisms, and pathophysiology. Neural and neuro-immune mechanisms of visceral hypersensitivity in irritable bowel síndrome. Am J Physiol Gastrointest Liver Physiol. (2012) 302:G1085–98. 10.1152/ajpgi.00542.2011 22403791 PMC3362095

[11] Jones RC III Otsuka E Wagstrom E Jensen CS Price MP Gebhart GF. Short-term sensitization of colon mechanoreceptors is associated with long-term hypersensitivity to colon distention in the mouse. Gastroenterology. (2007) 133:184–94. 10.1053/j.gastro.2007.04.042 17553498

[12] Bercik P Verdu EF Collins SM. Is irritable bowel syndrome a low-grade inflammatory bowel disease? Gastroenterol Clin North Am. (2005) 34:235–45. 10.1016/j.gtc.2005.02.007 15862932

[13] Gwee KA Collins SM Read NW Rajnakova A Deng Y Graham JC. Increased rectal mucosal expression of interleukin 1beta in recently acquired post-infectious irritable bowel syndrome. Gut. (2003) 52:523–6. 10.1136/gut.52.4.523 12631663 PMC1773606

[14] Kirsch R Riddell RH. Histopathological alterations in irritable bowel syndrome. Mod Pathol. (2006) 19:1638–45. 10.1038/modpathol.3800704 17013373

[15] De Schepper HU Cremonini F Park MI Camilleri M. Opioids and the gut: pharmacology and current clinical experience. Neurogastroenterol Motil. (2004) 16:383–94. 10.1111/j.1365-2982.2004.00513.x 15305992

[16] Barboza JL Talley NJ Moshiree B. Current and emerging pharmacotherapeutic options for irritable bowel syndrome. Drugs. (2014) 74:1849–70. 10.1007/s40265-014-0292-7 25260888

[17] Thomas RH Luthin DR. Current and emerging treatments for irritable bowel syndrome with constipation and chronic idiopathic constipation: focus on prosecretory agents. Pharmacotherapy. (2015) 35:613–30. 10.1002/phar.1594 26016701

[18] Khoshoo V Armstead C Landry L. Effect of a laxative with and without tegaserod in adolescents with constipation predominant irritable bowel syndrome. Aliment Pharmacol Ther. (2006) 23:191–6. 10.1111/j.1365-2036.2006.02705.x 16393297

[19] Grover M Drossman DA. Functional abdominal pain. Curr Gastroenterol Rep. (2010) 12:391–8. 10.1007/s11894-010-0125-0 20694840

[20] Drossman DA. Functional abdominal pain syndrome. Clin Gastroenterol Hepatol. (2004) 2:353–65. 10.1016/S1542-3565(04)00118-1 15118972

[21] Lea R Whorwell PJ. Quality of life in irritable bowel syndrome. Pharmacoeconomics. (2001) 19:643–53. 10.2165/00019053-200119060-00003 11456212

[22] Grossi V Hyams JS Glidden NC Knight BE Young EE. Characterizing clinical features and creating a gene expression profile associated with pain burden in children with inflammatory bowel disease. Inflamm Bowel Dis. (2019) 26:1283–1290. 10.1093/ibd/izz240 31627210

[23] Henstrom M D'Amato M. Genetics of irritable bowel syndrome. Mol Cell Pediatr. (2016) 3:7. 10.1186/s40348-016-0038-6 26873717 PMC4752571

[24] Zempsky WT O'Hara EA Santanelli JP Palermo TM New T Smith-Whitley K. Validation of the sickle cell disease pain burden interview-youth. J Pain. (2013) 14:975–82. 10.1016/j.jpain.2013.03.007 23701707 PMC3759569

[25] Salamon KS Davies WH Fuentes MR Weisman SJ Hainsworth KR. The pain frequency-severity-duration scale as a measure of pain: preliminary validation in a pediatric chronic pain sample. Pain Res Treat. (2014) 2014:653592. 10.1155/2014/653592 24579046 PMC3918349

[26] Crombez G Bijttebier P Eccleston C Mascagni T Mertens G Goubert L. The child version of the pain catastrophizing scale (PCS-C): a preliminary validation. Pain. (2003) 104:639–46. 10.1016/S0304-3959(03)00121-0 12927636

[27] Ebesutani C Reise SP Chorpita BF Ale C Regan J Young J. The Revised child anxiety and depression scale-short version: scale reduction via exploratory bifactor modeling of the broad anxiety factor. Psychol Assess. (2012) 24:833–45. 10.1037/a0027283 22329531

[28] Walker LS Beck JE Garber J Lambert W. Children's Somatization Inventory: psychometric properties of the revised form (CSI-24). J Pediatr Psychol. (2009) 34:430–40. 10.1093/jpepsy/jsn093 18782857 PMC2722132

[29] Jacob E Mack AK Savedra M Van Cleve L Wilkie DJ. Adolescent pediatric pain tool for multidimensional measurement of pain in children and adolescents. Pain Manag Nurs. (2014) 15:694–706. 10.1016/j.pmn.2013.03.002 23870767 PMC3808485

[30] Lavigne JV Schulein MJ Hahn YS. Psychological aspects of painful medical conditions in children II personality factors, family characteristics and treatment. Pain. (1986) 27:147–69. 10.1016/0304-3959(86)90207-1 3540811

[31] Perquin CW Hazebroek-Kampschreur A Hunfeld JAM Bohnen AM van Sujilekom-Smit LWA Passchier J. Pain in children and adolescents: a common experience. Pain. (2000) 87:51–8. 10.1016/S0304-3959(00)00269-4 10863045

[32] Kim YS Kim N. Sex-gender differences in irritable bowel syndrome. J Neurogastroenterol Motil. (2018) 24:544–58. 10.5056/jnm18082 30347934 PMC6175559

[33] Camilleri M. Sex as a biological variable in irritable bowel syndrome. Neurogastroenterol Motil. (2020) 32:e13802. 10.1111/nmo.13802 31943595 PMC7319890

[34] Deiteren A de Wit A van der Linden L De Man JG Pelckmans PA De Winter BY. Irritable bowel syndrome and visceral hypersensitivity: risk factors and pathophysiological mechanisms. Acta Gastroenterol Belg. (2016) 79:29–38. 26852761

[35] Zhou Q Verne GN. New insights into visceral hypersensitivity–clinical implications in IBS. Nat Rev Gastroenterol Hepatol. (2011) 8:349–55. 10.1038/nrgastro.2011.83 21643039 PMC3437337

[36] Chidambaran V Ashton M Martin LJ Jegga AG. Systems biology-based approaches to summarize and identify novel genes and pathways associated with acute and chronic postsurgical pain. J Clin Anesth. (2020) 62:109738. 10.1016/j.jclinane.2020.109738 32058259 PMC7276001

[37] Lemke JR Geider K Helbig KL. Delineating the GRIN1 phenotypic spectrum: A distinct genetic NMDA receptor encephalopathy. Neurology. (2016) 86:2171–8. 10.1212/WNL.0000000000002740 27164704 PMC4898312

[38] Yuan H Low CM Moody OA Jenkins A Traynelis SF. Ionotropic GABA and glutamate receptor mutations and human neurologic diseases. Mol Pharmacol. (2015) 88:203–17. 10.1124/mol.115.097998 25904555 PMC4468639

[39] Yuan H Hansen KB Zhang J. Functional analysis of a de novo GRIN2A missense mutation associated with early-onset epileptic encephalopathy. Nat Commun. (2014) 5:3251. 10.1038/ncomms4251 24504326 PMC3934797

[40] Yi F Bhattacharya S Thompson CM Traynelis SF Hansen KB. Functional and pharmacological properties of triheteromeric GluN1/2B/2D NMDA receptors. J Physiol. (2019) 597:5495–514. 10.1113/JP278168 31541561 PMC6858497

[41] Chaudhry UA Dore S. Cytosolic prostaglandin E synthase: expression patterns in control and Alzheimer's disease brains. Am J Alzheimers Dis Other Demen. (2009) 24:46–51. 10.1177/1533317508323655 19001348 PMC2859688

[42] Echeverria V Clerman A Dore S. Stimulation of PGE receptors EP2 and EP4 protects cultured neurons against oxidative stress and cell death following beta-amyloid exposure. Eur J Neurosci. (2005) 22:2199–206. 10.1111/j.1460-9568.2005.04427.x 16262658

[43] Sarkar S Hobson AR Hughes A. The prostaglandin E2 receptor-1 (EP-1) mediates acid-induced visceral pain hypersensitivity in humans. Gastroenterology. (2003) 124:18–25. 10.1053/gast.2003.50022 12512025

[44] Breyer RM Bagdassarian CK Myers SA Breyer MD. Prostanoid receptors: subtypes and signaling. Annu Rev Pharmacol Toxicol. (2001) 41:661–90. 10.1146/annurev.pharmtox.41.1.661 11264472

[45] Herman JA Willits AB Bellemer A. Galphaq and Phospholipase Cbeta signaling regulate nociceptor sensitivity in Drosophila melanogaster larvae. PeerJ. (2018) 6:e5632. 10.7717/peerj.5632 30258723 PMC6151255

[46] Lai S Pelech S. Regulatory roles of conserved phosphorylation sites in the activation T-loop of the MAP kinase ERK1. Mol Biol Cell. (2016) 27:1040–50. 10.1091/mbc.E15-07-0527 26823016 PMC4791125

[47] Zhang Y Huang X Wang J Wang X Liu X Chen Y. Nitration-induced ubiquitination and degradation control quality of ERK1. Biochem J. (2019) 476:1911–26. 10.1042/BCJ20190240 31196894 PMC6604951

[48] Korczeniewska OA Katzmann Rider G Gajra S Narra V Ramavajla V Chang Y-j. Differential gene expression changes in the dorsal root versus trigeminal ganglia following peripheral nerve injury in rats. Eur J Pain. (2020) 24:967–82. 10.1002/ejp.1546 32100907

[49] Li XL Zhou CY Sun Y Su ZY Wang X Jia EN. Bioinformatic analysis of potential candidates for therapy of inflammatory bowel disease. Eur Rev Med Pharmacol Sci. (2015) 19:4275–84. 26636514

[50] Layhadi JA Fountain SJ. P2X4 Receptor-Dependent Ca(2+) Influx in Model Human Monocytes and Macrophages. Int J Mol Sci. (2017) 18:2261. 10.3390/ijms18112261 29077063 PMC5713231

[51] Layhadi JA Turner J Crossman D Fountain SJ. ATP evokes Ca(2+) responses and CXCL5 secretion via P2X4 receptor activation in human monocyte-derived macrophages. J Immunol. (2018) 200:1159–68. 10.4049/jimmunol.1700965 29255078 PMC5784824

[52] Bo X Kim M Nori SL Schoepfer R Burnstock G North RA. Tissue distribution of P2X4 receptors studied with an ectodomain antibody. Cell Tissue Res. (2003) 313:159–65. 10.1007/s00441-003-0758-5 12845522

[53] Ulmann L Hirbec H Rassendren F. P2X4 receptors mediate PGE2 release by tissue-resident macrophages and initiate inflammatory pain. EMBO J. (2010) 29:2290–300. 10.1038/emboj.2010.126 20562826 PMC2910276

